# Sorrowing Poverty

**Published:** 1859-04

**Authors:** 


### SORROWING POVERTY.

THAT infant children should be starved to death deliberately in the great city of New York, is an almost incredible statement, except to the few, whose large intercourse with the world, has led them to the observation, that there is no meanness so unfathomable but some human wretch may be found who shall dive down and perpetrate it.

On a February day, within six squares of the palatial residences of Fifth Avenue, three infants were found, so abject and idiotic in expression that all trace of humanity seemed lost. "They could not cry, and so brutish were they, that when lifted from their cradles they merely gazed about, as puppies or kittens; none of them had any flesh on their bones."

Nearly two thousand children whose parents cannot take care of them, are constantly on the hands of the city; the helpless children of crime, of poverty, of infidelity and prostitution. About two hundred of the above are to be nursed, and are distributed over the city to women who profess to have lost their own children; of these two hundred, were the three above referred to.

We do not hold up to public reprobation the woman who engaged to take care of these three children, at the stipulated payment of one dollar a week each, the price paid for boarding dogs in —— street, for most likely she was poor and ignorant and perhaps herself at not a great remove from starvation.—Half frozen and famishing, the very best of us can't say what we would not do. Theoretical incorruptibility is the easiest of all easy things, to those who roll in wealth.

Ten men are elected, called governors of the alms house. It is a position of honor and responsibility, for hundreds of thousands of dollars pass through their hands every year. These governors will not let a child go out to nurse until a respectable physician certifies that the woman is every way proper to receive the child; next, a visitor is sent to the house to see if appearances correspond, and if so the child is given out, and the matron of the alms house is required to visit each child once or twice a month. And yet, notwithstanding all this, here are three children accidentally found in the condition above named. These statements simply show the unfitness for office, somewhere.

Three-fourths of all children picked up in the streets of New York, die before their teens, because they are born diseased. In cases where most especial care was given, two-thirds died, showing this, that the children of vice are born diseased, are brought into the world with the physical maladies of their parents. Three-fourths of the idiotic in a Massachusetts charity, were found to be of parents, one or both of whom were drunken. Physical vices therefore are not only perpetuated in the offspring, but they originate mental deformities. But if this is true of the degraded, it must be true of the genteely vicious. A Fifth Avenue mother, who indulges in opium or wines or cordials, is just as likely to have an idiotic child, as the besotted of the purlieus. The gourmand of Fourteenth Street and Madison Square, who is always "full," in skin and paunch as well as in purse, is just as likely to have a child which shall perish with marasmus or chronic diarrhœa, as the other of bad liquors at the five points.

Therefore, those who do not make good health a study and an aim, who do not practice daily the temperances and self denials which seldom fail to secure this good health, are committing a crime against their unborn children, which they never can atone for.

